# What the algorithm serves: diet and fitness content on Instagram as independent predictors of orthorexia nervosa in a large Italian female sample

**DOI:** 10.3389/fnut.2026.1937445

**Published:** 2026-09-23

**Authors:** Edoardo Mocini, Francesca Greco, Arianna Gagliardi, Luca Titi, Elisa Giannetta, Maria Grazia Tarsitano

**Affiliations:** 1 Department of Theoretical and Applied Sciences, eCampus University, Novedrate, Italy; 2 Department of Experimental and Clinical Medicine, Magna Graecia University of Catanzaro, Catanzaro, Italy; 3 Department of Experimental Medicine, Sapienza University of Rome, Rome, Italy; 4 Department of Emergency, Critical Care, and Trauma, Policlinico Umberto I, Rome, Italy; 5 Department of Human Sciences and Promotion of the Quality of Life, San Raffaele Roma Open University, Rome, Italy

**Keywords:** cross-sectional, dietary content, Instagram, orthorexia nervosa, social media

## Abstract

**Objective:**

Instagram use has been associated with orthorexia nervosa (ON), but prior research has generally measured exposure as overall platform use or broad health-related content. We directly compared four content categories (diet and slimming products, physical activity and sport, food in general, and fashion) within one adjusted model and provided an exploratory description of Instagram use and orthorexic symptoms in women with fibromyalgia.

**Methods:**

Cross-sectional web-based survey of 3,655 Italian adult women using Instagram as their primary social media platform. Content exposure was assessed on a 0–4 ordinal scale. Multivariable linear regression with HC3 robust standard errors was applied after z-scoring all predictors. A sensitivity analysis added daily Instagram use time.

**Results:**

Diet and slimming content (B = +1.14, 95% CI [+0.91, +1.38]; *p* < 0.001) and sport content (B = +0.91, 95% CI [+0.69, +1.12]; *p* < 0.001) were the strongest independent predictors of I-DOS scores. General food content was also independently associated (B = +0.50; *p* < 0.001), whereas fashion content was not (B = +0.003; *p* = 0.97). Time on platform was not independently associated after content adjustment (Δ*R*^2^ = 0.0006). No content difference survived Bonferroni correction in women with fibromyalgia (*n* = 37), and their I-DOS scores did not differ significantly.

**Discussion:**

The Instagram–ON association was content-specific: diet and fitness content, rather than fashion content or time on the platform, accounted for the pattern. Because the data are cross-sectional and self-reported, causal direction cannot be established.

## 1 Introduction

Instagram has become a routine setting for social connection, self-presentation, and health information for many users. Its image-based feed and personalised recommendations can repeatedly place diet, wellness, and fitness messages in front of the same user (1, 2).

Orthorexia nervosa (ON) is characterised by a strong preoccupation with one’s eating behaviour and self-imposed rigid, inflexible dietary rules that are strictly controlled, accompanied by marked distress upon their violation, and associated with progressive impairment of social and occupational functioning (3). Although ON is not formally classified in the DSM-5-TR or ICD-11, a multi-society consensus definition and proposed diagnostic criteria have been developed based on structured expert agreement (3), and the condition has attracted substantial clinical and research attention due to its functional consequences and its overlap with obsessive-compulsive and other eating disorder spectra (4–6).

Prevalence estimates vary considerably across studies (6.9%–88.7%), primarily due to heterogeneity in assessment instruments and population samples (7). Young women have been found to exhibit positive correlations between I-DOS scores and body mass index, physical activity levels, and sexual attitudes, while no such associations have emerged in men (8). Among validated tools for ON assessment in Italian-speaking populations, the Italian-Dusseldorf Orthorexia Scale (I-DOS) and the Orthorexia Nervosa Inventory (ONI) have demonstrated good psychometric properties (9, 10). Epidemiological evidence consistently identifies specific vulnerability groups, including women, individuals with high educational attainment, athletes, and practitioners of restrictive dietary patterns including vegans and vegetarians (6, 11, 12). Psychosocial risk factors include perfectionism, anxiety, obsessive-compulsive traits, body image concerns, and social norms around healthy eating (12).

These features have made Instagram the social media platform most consistently implicated in ON. Large communities organised around clean eating, wellness, and fitness may amplify orthorexic cognitions and behaviours (1, 2). Multiple cross-sectional studies have documented positive associations between Instagram use and orthorexia symptom scores (1, 2, 13, 14), and a large Italian survey of Instagram users found that time spent on social media was an independent predictor of orthorexic symptoms (15). A recent scoping review of 31 studies found that health-related dietary and fitness content was the dimension most consistently associated with ON symptoms, while non-specific platform use showed weaker and less consistent associations (16).

However, prior research has often treated Instagram exposure as a single variable, usually time spent on the platform or broad engagement with health-related content, without comparing several content categories in the same adjusted model. This makes it difficult to identify which type of content may matter most. Qualitative and observational evidence suggests that diet and fitness content may promote dietary restriction norms and food purity beliefs that closely resemble orthorexic cognitions (13, 16). Fashion content may act through a different route, mainly body comparison and thin-ideal internalisation. Comparing these content streams directly is therefore relevant to both explanation and intervention.

A secondary question of clinical interest is whether specific patient groups may show different patterns of exposure to orthorexia-relevant Instagram content. Fibromyalgia is a chronic widespread pain syndrome predominantly affecting women (17, 18), associated in some patients with intensive health-information seeking and dietary self-management, including anti-inflammatory and elimination diets adopted without clinical guidance. Women with fibromyalgia who search for health-related content on Instagram may be more likely to encounter dietary restriction material and may show elevated orthorexic symptom burden as a result of this clinical and behavioural profile. Because the present study did not measure objective algorithmic exposure or content-seeking behaviour, this analysis is explicitly exploratory.

The main novel contribution of this study is the simultaneous comparison of four content categories in one adjusted model, allowing content type to be considered separately from overall time spent on Instagram. Accordingly, the study had two objectives: first, to test whether diet and fitness content were more strongly associated with I-DOS scores than fashion content; and second, to provide an exploratory description of Instagram content exposure and orthorexic symptoms in women with fibromyalgia.

## 2 Materials and methods

### 2.1 Study design and participants

We conducted a cross-sectional web-based survey among Italian adults in February 2022, distributed anonymously via social media channels and promoted through word-of-mouth among Instagram users. All participants provided informed consent. The study complied with the Declaration of Helsinki (2013) and the General Data Protection Regulation (GDPR 679/2016). Inclusion criteria were age greater than 18 years and the ability to complete the survey independently.

Of the 4,113 total respondents, 191 men and individuals of other genders were excluded, leaving 3,922 women. Of these, 258 who did not report Instagram as their primary social media platform were excluded, leaving 3,664. A further 9 records were excluded due to implausible or missing BMI values (outside the range 12–70 kg/m^2^). The final analytic sample comprised 3,655 women.

### 2.2 Measures

#### 2.2.1 Orthorexia nervosa assessment

ON was assessed using the I-DOS (9), the Italian adaptation of the Dusseldorf Orthorexia Scale. The instrument comprises 10 items evaluating orthorexic attitudes and behaviours, each rated on a four-point Likert scale from 1 (“this does not apply to me at all”) to 4 (“this applies to me completely”), yielding a total score from 10 to 40; higher scores indicate a greater orthorexic symptom burden. Internal consistency in the present sample was Cronbach’s α = 0.871 (item-total Spearman correlations: 0.41–0.69); McDonald’s ω = 0.876. The I-DOS distribution was right-skewed (skewness = 0.96). The Shapiro–Wilk test was used only as a descriptive check on a random subsample of n = 200 (*p* < 0.001), because with N = 3,655 it would be highly sensitive to small, practically unimportant departures from normality. No observations were excluded on the basis of this test. The I-DOS total score was the primary continuous outcome.

#### 2.2.2 Instagram content exposure

Participants reported how often four categories of content appeared in their Instagram feed: (i) diet products and slimming aids, (ii) physical activity and sport, (iii) food in general, and (iv) fashion images. Responses were recorded on a five-point ordinal scale from 0 (“almost none or none”) to 4 (“almost all content”). For each content category, women with no exposure (score = 0) were compared against women with high exposure (score ≥ 3) to characterise the clinical relevance of differential exposure.

#### 2.2.3 Additional variables

Demographic covariates were age (years), BMI (kg/m^2^, derived from self-reported weight and height; values outside 12–70 kg/m^2^ set to missing; one entry with height recorded as 1.50 instead of 150 cm was corrected), and education level (ordinal scale: 1 = lower secondary; 2 = upper secondary diploma; 3 = arts/music conservatory; 4 = bachelor’s degree; 5 = master’s degree; 6 = doctoral degree). Daily Instagram use was assessed on a four-category ordinal scale (less than 15, 15–30, 31–60, more than 60 min). Self-reported fibromyalgia was coded as binary (yes/no).

### 2.3 Statistical analysis

Continuous variables are presented as mean and standard deviation (SD) or median and interquartile range (IQR); categorical variables as counts and percentages.

Spearman rank correlations were computed between each content exposure variable and I-DOS score, given the non-normal outcome distribution and ordinal nature of the exposure variables. For each content category, Mann–Whitney U tests compared I-DOS scores between no-exposure (score = 0) and high-exposure (score ≥ 3) groups. Because Kolmogorov–Smirnov tests indicated that the I-DOS distributions differed in shape as well as location for all content categories (all KS *p* < 0.001), the Mann–Whitney U was interpreted as a test of distributional difference and probabilistic superiority rather than as a simple difference of medians. Effect sizes were expressed as Cohen’s d (pooled standard deviation) and as the rank-biserial correlation r (native effect size of the Mann–Whitney U; *r* = 1 − 2 U/[n₁ × n₂]).

A multivariable linear regression model was specified with I-DOS score as the outcome and all four content variables, age, BMI, and education level as predictors. Ordinal exposure and covariate variables were treated as numeric scores and all model predictors were standardised (z-scored) to allow direct comparison of coefficients. Given the large sample size, regression estimates were expected to have acceptable large-sample inferential properties; however, significant heteroscedasticity was detected (Breusch-Pagan LM = 270.1, *p* < 0.001).

Accordingly, HC3 robust standard errors were used for all confidence intervals and t statistics. Variance inflation factors (VIF) were computed to assess multicollinearity. As a planned sensitivity analysis, daily Instagram use time (z-scored) was added to the primary regression model to test whether content associations were independent of overall platform use time. Tarsitano et al. (15) previously established, in the full dataset from this survey, that time on social media independently predicted ON symptom burden; the present analysis tests whether content-specific associations persist after accounting for that predictor.

Between-group differences in Instagram content exposure and I-DOS scores between women with and without fibromyalgia were assessed using Mann–Whitney U tests. Given the small case group (*n* = 37), Bonferroni correction was applied for four simultaneous content comparisons (corrected threshold: *p* < 0.0125). Results are reported descriptively as hypothesis-generating.

Before the primary analyses, assumptions and diagnostics were evaluated. Normality was assessed descriptively with the Shapiro–Wilk test on a random subsample (*n* = 200; W = 0.928, *p* < 0.001). The subsample limited the test’s sensitivity to trivial departures from normality in a large dataset; it did not determine inclusion or the regression analysis, both of which used all 3,655 observations. The observed right skew and the ordinal exposure measures supported the use of Spearman correlations and Mann–Whitney U tests in the bivariate analyses. The Ramsey RESET test indicated no evidence of functional-form misspecification (*F* = 2.341, *p* = 0.096). The maximum VIF was 1.45 (sport content), indicating no problematic collinearity. Using the screening threshold 4/N = 0.0011, 234 observations (6.4%) exceeded the Cook’s distance threshold; the maximum value was 0.0085, well below 1.0. Excluding these observations did not change the primary conclusions. Analyses followed the IJED Statistical Reporting Guidelines.

## 3 Results

### 3.1 Sample characteristics

The analytic sample comprised 3,655 Italian women who reported Instagram as their primary social media platform. Mean age was 30.7 years (SD 8.5); mean BMI 24.3 kg/m^2^ (SD 5.5). The majority held at least a bachelor’s degree (66.5%). A total of 41.6% spent more than 60 min per day on Instagram. The mean I-DOS score was 19.4 (SD 5.7; median 18.0, IQR [15–22]). Full characteristics are shown in Table 1.

**Table 1:** Characteristics of the analytic sample (*N* = 3,655 Italian women using Instagram as their primary social media platform).

Characteristic	*n* (%)	Mean (SD) or median [IQR]
Total analytic sample	3,655 (100%)	
Age, years		30.7 (8.5)
BMI, kg/m^2^		24.3 (5.5)
Education level^1^		4 [2–5]
I-DOS score (range 10–40)		19.4 (5.7)
Daily Instagram use		
<15 min	183 (5.0%)	
15–30 min	564 (15.4%)	
31–60 min	1,389 (38.0%)	
> 60 min	1,519 (41.6%)	
Instagram content exposure (0–4)^2^		
Diet and slimming products		0.88 (1.06)
Physical activity and sport		1.62 (1.07)
Food (general)		2.20 (0.96)
Fashion		1.30 (1.15)
Self-reported fibromyalgia	37 (1.0%)	

1 1 = lower secondary school through 6 = doctoral degree; median and IQR reported.

2 0 = almost none or none; 4 = almost all content.

### 3.2 Spearman correlations

All four content exposure variables were positively and significantly correlated with I-DOS scores. The strongest bivariate correlations involved sport and physical activity content (*ρ* = +0.266, *p* < 0.001) and diet and slimming content (*ρ* = +0.260, *p* < 0.001), followed by food content (*ρ* = +0.203, *p* < 0.001). Fashion content showed a substantially weaker correlation (*ρ* = +0.081, *p* < 0.001). Results are shown in Table 2.

**Table 2:** Spearman rank correlations between Instagram content exposure and I-DOS score (*N* = 3,655).

Content category	Mean (SD)	High exposure^1^ (%)	Spearman *ρ*	*p*
Diet and slimming products	0.88 (1.06)	7.6%	+0.260	<0.001
Physical activity and sport	1.62 (1.07)	15.5%	+0.266	<0.001
Food (general)	2.20 (0.96)	28.2%	+0.203	<0.001
Fashion	1.30 (1.15)	13.2%	+0.081	<0.001

1 Percentage of women with frequent or very frequent exposure (score ≥ 3 on the 0–4 ordinal scale). All *p*-values < 0.001.

### 3.3 Exposure-stratified I-DOS comparisons

Women with frequent diet and slimming content exposure (score ≥ 3; *n* = 276) had I-DOS scores 4.98 points higher than those with no such exposure (score = 0; *n* = 1,808): mean 22.86 (SD 7.27) vs. 17.88 (SD 4.75); Mann–Whitney U, *p* < 0.001; Cohen’s d = 0.81, rank-biserial *r* = 0.41. For sport and physical activity content, the difference was 5.21 points (22.48 vs. 17.27; Cohen’s d = 0.89, rank-biserial *r* = 0.46; *p* < 0.001). For general food content, 4.23 points (21.14 vs. 16.91; Cohen’s d = 0.73, rank-biserial *r* = 0.40; *p* < 0.001).

For fashion content, frequent exposure (score ≥ 3; *n* = 483) was associated with a 1.90-point higher I-DOS score relative to no exposure (score = 0; *n* = 1,138): mean 20.70 (SD 6.43) vs. 18.80 (SD 5.32); Mann–Whitney U, *p* < 0.001; Cohen’s d = 0.32, rank-biserial *r* = 0.17. This difference is statistically significant but substantially smaller than those observed for dietary and sport content, and as the multivariable model demonstrates, it is entirely explained by confounding with the other content variables.

### 3.4 Multivariable regression

The multivariable model (*N* = 3,655; *R*^2^ = 0.130; adjusted *R*^2^ = 0.128; *F*(7, 3,647) = 56.83; all VIF ≤ 1.45) showed that diet and slimming content was the strongest independent content predictor of I-DOS scores (B = +1.144, 95% CI [+0.909, +1.378]; *p* < 0.001), followed by sport and physical activity content (B = +0.905, 95% CI [+0.688, +1.121]; *p* < 0.001) and general food content (B = +0.502, 95% CI [+0.301, +0.703]; *p* < 0.001). Fashion content showed no independent association (B = +0.003, 95% CI [−0.186, +0.193]; *p* = 0.973). Because predictors were z-scored while I-DOS remained in its original units, B is the expected change in I-DOS points per 1-SD increase in a predictor. Among demographic covariates, only BMI was independently associated (B = −0.520; *p* < 0.001), with higher BMI predicting lower I-DOS scores. Age and education level showed no independent associations. Full results are in Table 3.

**Table 3:** Multiple linear regression predicting I-DOS score (*N* = 3,655 Instagram users).

Predictor	B (per 1 SD)	Robust SE	*t*	*p*	95% CI for B
Diet/slimming content	+1.144	0.120	+9.55	<0.001	[+0.909, +1.378]
Sport and physical activity	+0.905	0.110	+8.19	<0.001	[+0.688, +1.121]
Food content (general)	+0.502	0.103	+4.89	<0.001	[+0.301, +0.703]
Fashion content	+0.003	0.097	+0.03	0.973	[−0.186, +0.193]
BMI (kg/m^2^)	−0.520	0.091	−5.74	<0.001	[−0.697, −0.343]
Age (years)	−0.075	0.090	−0.83	0.405	[−0.252, +0.102]
Education level	−0.056	0.091	−0.61	0.541	[−0.235, +0.123]

Predictors were z-scored; B is the expected change in I-DOS points for a 1-SD increase in the predictor. Robust standard errors and confidence intervals use the HC3 estimator because the Breusch-Pagan test indicated unequal residual variance (LM = 270.1, *p* < 0.001). Model summary: *R*^2^ = 0.130; adjusted *R*^2^ = 0.128; F(7, 3,647) = 56.83; maximum VIF = 1.45.

### 3.5 Sensitivity analysis with time on platform

When daily Instagram use time was added to the primary model, content coefficients were essentially unchanged (diet: B = +1.140; sport: B = +0.895; food: B = +0.502; fashion: B = +0.006). Time on platform was not independently associated with I-DOS after content adjustment (B = −0.143, 95% CI [−0.323, +0.038]; *p* = 0.121; Δ*R*^2^ = 0.0006). Content-specific associations were therefore not explained by overall platform use time.

### 3.6 Fibromyalgia exploratory analysis

Of the 3,655 Instagram users in the analytic sample, 37 (1.0%) reported a diagnosis of fibromyalgia. Women with fibromyalgia showed a non-significant trend toward higher I-DOS scores than those without (mean 20.9 ± 6.6 vs. 19.3 ± 5.7; Mann–Whitney U, *p* = 0.142; Cohen’s d = 0.26, rank-biserial *r* = 0.14; post-hoc power for d = 0.26: 35%). In terms of Instagram content exposure, no content category reached the Bonferroni-corrected significance threshold (*p* < 0.0125). Fashion content showed a nominally significant difference (mean 1.78 ± 1.27 vs. 1.30 ± 1.15; *p* = 0.020; SMD = +0.40) that did not survive correction. Diet content showed a positive but non-significant trend (mean 1.19 ± 1.22 vs. 0.87 ± 1.06; *p* = 0.089; SMD = +0.28). No significant differences were found for sport or food content (both *p* > 0.23). Given the small group size and the absence of findings surviving multiple comparison correction, these observations are exploratory and hypothesis-generating.

## 4 Discussion

Two findings stand out. First, diet and slimming content (B = +1.144) and fitness content (B = +0.905) were the strongest independent content predictors of I-DOS scores; general food content showed a smaller association (B = +0.502). Second, fashion content was no longer associated with I-DOS after the content categories were considered together (B = +0.003, *p* = 0.973), despite its weak bivariate correlation. The study’s novel contribution is this direct, mutually adjusted comparison: it suggests that what users see may be more informative than Instagram use alone. The pattern also agrees with a recent scoping review of 31 studies, in which dietary and fitness content was more consistently linked to ON symptoms than non-specific exposure (16).

The model explained 13.0% of I-DOS variance (*R*^2^ = 0.130). This is meaningful but modest, as orthorexic symptoms also reflect psychological, personality, and contextual factors that were not measured here. Importantly, adding daily Instagram use time did not alter the content coefficients (change in B < 0.01 for all), and time itself was not independently associated with I-DOS (B = −0.143, *p* = 0.121). This refines the earlier finding by Tarsitano et al. (15) that time on social media predicts ON in this cohort: once content is considered, content type may be more informative than time alone.

One result may seem surprising. Appearance-focused Instagram content is widely linked to eating-disorder risk through body comparison and dissatisfaction (1, 13), yet fashion content had a near-zero independent association in this study. Diet and fitness content showed much stronger associations. These results point more clearly to food- and health-focused messages than to appearance content in relation to ON. However, they cannot show whether rule-following, food-purity beliefs, ritualised health behaviour, or obsessive-compulsive processes account for the pattern (4).

The main unanswered question is causal direction. Three explanations fit the cross-sectional results. Diet and fitness content may reinforce orthorexic thoughts through repeated exposure; people with pre-existing orthorexic traits may seek out this material; or people with higher symptoms may simply notice and remember food-related posts more readily. The third explanation is especially relevant because exposure was reported by participants rather than measured from their feeds. If food-related stimuli are more salient to people with orthorexic characteristics (12, 19, 20), perceived exposure may partly reflect their symptoms. The present data cannot separate these explanations.

Future studies should therefore measure the posts actually delivered to each user, follow exposure and symptoms over time, or experimentally vary feed content. These designs could show whether intervention should focus on platform recommendations, users’ choices, or attention to food-related cues.

The general food content association (B = +0.502) was weaker than those for diet and fitness content, probably because this broad category includes both restriction-focused and neutral culinary posts. The inverse BMI-I-DOS association (B = −0.520) is consistent with prior literature and remained independent of content exposure (21).

The fibromyalgia analysis remains exploratory. With only 37 cases, the study had limited power to detect the observed I-DOS difference (d = 0.26, *p* = 0.142). The nominal fashion-exposure finding (*p* = 0.020) did not survive correction for multiple comparisons and should be used only to generate hypotheses for a larger dedicated study (17, 18).

### 4.1 Strengths and limitations

The study has four main strengths: a large sample, use of the validated I-DOS, direct comparison of four content categories in one adjusted model, and robust inference using HC3 standard errors and a Cook’s distance sensitivity analysis. Excluding 234 potentially influential observations did not change the conclusions: diet (B = +1.02), fitness (B = +0.89), and food content (B = +0.38) remained independently associated with I-DOS (all *p* < 0.001), whereas fashion content did not (*p* = 0.61).

The main limitations concern design and measurement. Because the study is cross-sectional, it cannot establish whether content exposure precedes symptoms. All primary variables were self-reported: BMI may be underestimated, and dietary patterns and clinical diagnoses, including fibromyalgia, were not clinically verified. Perceived content exposure deserves particular caution. Participants with higher I-DOS scores may notice diet-related material more readily, so the exposure measure may partly reflect symptom-related attention rather than differences in the posts delivered to them. No sensitivity analysis can remove this source of bias from the present data.

Other limitations affect generalisability and model specification. The ordinal exposure variables were modelled as linear scores, so threshold or non-linear associations may have been missed. Participants were not a clinical ON sample, no diagnostic cutoff was applied, and the self-selected sample overrepresented young, educated, highly active Italian Instagram users. The findings may not extend to men, other platforms, or less active users. The four content categories also omitted potentially relevant material such as wellness influencers, supplements, and clean-eating communities. Finally, data were collected in February 2022, when pandemic-related patterns may have kept social media use above usual levels (22).

### 4.2 Clinical implications and future directions

The practical message is specific but limited: diet, slimming, and fitness content may be more useful markers of orthorexic symptoms than fashion content or time spent on Instagram. The cross-sectional results do not show that Instagram recommendations caused the symptoms. For prevention and clinical assessment, asking what content a person follows or recalls may nevertheless be more informative than asking only how long they use the platform. Future research should combine objective feed data with longitudinal or experimental designs to determine whether the observed pattern reflects algorithmic recommendations, users’ choices, or symptom-related attention.

## Data Availability

The raw data supporting the conclusions of this article will be made available by the authors, without undue reservation.
